# A New Look at Older Adults’ Use of Ride Share Services: Insights from a National Survey

**DOI:** 10.1093/geroni/igaf122.2010

**Published:** 2025-12-31

**Authors:** Alycia Bayne, Alexa Siegfried, Lindsey Witt-Swanson

**Affiliations:** National Opinion Research Center at The University of Chicago, Chicago, Illinois, United States; National Opinion Research Center at The University of Chicago, Chicago, Illinois, United States; National Opinion Research Center at The University of Chicago, Chicago, Illinois, United States

## Abstract

Ride share services are a promising transportation solution for older adults. While research has documented that ride share use among older adults has generally increased since 2015, no studies have examined national trends in their use among older adults across age groups and by geography. To address this research gap, NORC conducted a nationally representative survey of 1,010 U.S. adults aged 50+ using the NORC Foresight 50+ Panel, which is drawn from the NORC national sample frame that has over 97% coverage of U.S. addresses. The survey was fielded in May 2024 and included questions about ride share use, trip purpose, frequency of use, and factors affecting use. This session describes findings from our descriptive analysis to identify the characteristics of older adults who had ever used a ride share service, including age, education, and income, trip purpose, frequency of use, and factors affecting use. We also examined variation in the availability of ride share services. This session describes our findings, including that nearly half of older adults (45.6%) reported using a ride share service, with most using it monthly or less (92.8%). Geographic differences in use also emerged. Factors affecting use include convenience, safety, and affordability. This study provides a clear picture of the characteristics of older adults who use ride share services. Further, this study has implications for public health practitioners and transportation planners developing transportation programs that meet the health and mobility needs of older adults.

